# Determining the effect of different environmental conditions on Ebola virus viability in clinically relevant specimens

**DOI:** 10.1038/s41426-018-0043-z

**Published:** 2018-03-29

**Authors:** Bernadett Palyi, Nora Magyar, Judit Henczko, Balint Szalai, Agnes Farkas, Thomas Strecker, Maria Takacs, Zoltan Kis

**Affiliations:** 1 National Biosafety Laboratory, National Public Health Institute, Budapest, 1097 Hungary; 2 Division of Virology, National Public Health Institute, Budapest, 1097 Hungary; 3 Institute of Virology, Philipps University Marburg, Marburg, 35043 Germany; 4 Institute of Medical Microbiology, Semmelweis University, Budapest, 1089 Hungary

## Abstract

In 2013–2016, West Africa experienced the largest and longest Ebola virus disease outbreak ever documented. The wide geographic spread and magnitude of the outbreak often limited the timely and rapid testing of diagnostic samples from patients with suspected Ebola virus disease, raising questions regarding the optimal storage and shipping conditions of clinically relevant specimens, including EDTA-whole blood, plasma, capillary blood, urine and seminal fluid (associated with sexual transmission of the Ebola virus after recovery from the disease). Therefore, the aim of our study was to identify the extent to which storage temperature and clinical specimen type influence Ebola virus viability. Virus infectivity was determined using a fluorescent focus-forming assay. In our study, we show that Ebola virus was the most stable in EDTA-whole blood and plasma samples, whereas rapid decay of infectivity was observed in simulated capillary blood, urine and semen samples, especially when these samples were stored at higher temperatures. The analysis of variance results demonstrated that both temperature and clinical specimen type have significant effects on virus viability, whereas donor differences were not observed. Repeated freeze and thaw cycles of the samples also had a notable impact on virus viability in EDTA-whole blood and urine. Due to the rapid temperature- and specimen-dependent degradation of the virus observed here, our study highlights the importance of proper clinical sample storage at low temperatures during transportation and laboratory analysis.

## Introduction

Ebola outbreaks, especially those that occurred in 2013–2016 in West Africa, pose significant challenges for outbreak response teams with respect to ensuring adequate conditions for the collection, transport, storage and processing of different biological specimens for laboratory testing^1^. According to the recommendations of the World Health Organization and the Pan American Health Organization, samples can be refrigerated (2–8 °C) for up to a week. However, shipping of the samples to reference laboratories during the first 48 h after collection is highly recommended and may influence test reliability^2^. The RNA of Ebola virus (EBOV) is known to be relatively stable in whole blood and serum/plasma samples for weeks under African environmental conditions, depending on the initial viral load^3–5^. However, alternative sample types, including oral swabs and capillary blood samples, are also evaluated for nucleic acid-based diagnosis, as are urine and semen samples from which EBOV can be detected by PCR for several weeks or even months after symptoms onset^3,6–8^. The environmental stability of the virus has also been studied, and the potential persistence of its viral RNA under hospital circumstances has been determined^9,10^. EBOV RNA in Ebola treatment centers was found to be associated with surfaces that come into close contact with patients and infectious body fluids, such as caregiver gloves, beds and blankets^10^. Studies confirmed that EBOV maintains infectivity in liquid blood samples for a longer period of time than in dried blood but that relative humidity and temperature can also impact virus viability^4,9,11^. While adequate storage conditions of clinical specimens for nucleic acid-based diagnosis are well studied, knowledge regarding the stability of infectious EBOV in different clinical specimens stored under various conditions is limited. Such information is critical in order to obtain reliable data about the potential infectiousness of secretions from patients with Ebola virus disease (EVD). Several longitudinal studies have also addressed the persistence of EBOV in different body fluids, such as the seminal fluids of male survivors. This information is critical to assess the risk of transmission of EBOV from survivors within the community^12–15^. However, poor transport and storage conditions of clinical diagnostic samples can reduce virus viability and complicate infectivity studies. Identifying the effect of inappropriate storage conditions is critical for confirming the reliability of longitudinal persistence studies by virus isolation. Therefore, our aim was to determine the viability of the EBOV strain Makona during short-term storage in different clinical sample specimens, such as EDTA-whole blood, plasma, simulated capillary blood, urine and semen, under a range of simulated African environmental conditions.

## Results

**Fig. 1:**
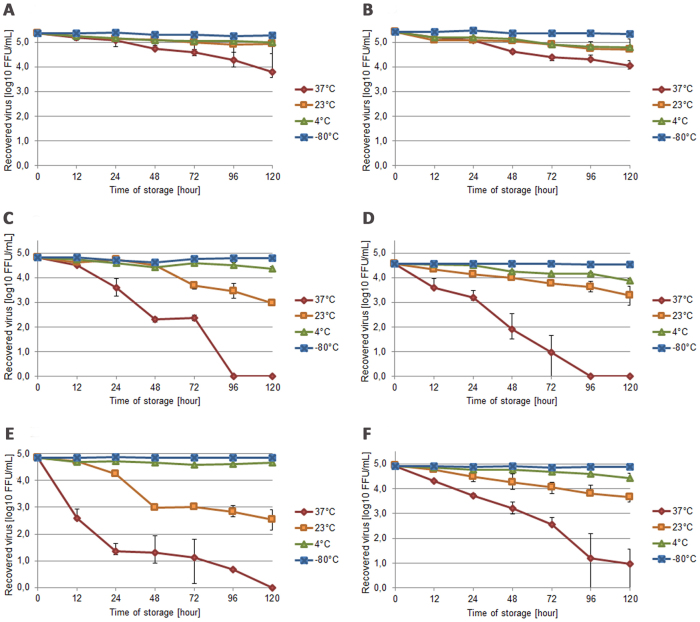
Infectivity of Ebola virus during short-term storage in different clinical specimens. **a** EDTA-whole blood, **b** plasma, **c** simulated capillary blood, **d** urine, **e** semen and **f** RPMI-cell culture media (control) at different temperatures. The data points represent the mean values calculated from the average log10 FFU/mL reduction in each of the volunteers (error bars show SD values). Each sample was titrated in triplicate

EBOV viability during short-term storage was tested in different clinically relevant specimen types, such as EDTA-whole blood, plasma, simulated capillary blood, as well as urine and semen. RPMI-1640 cell culture medium was used as a control. The samples, which were obtained from healthy volunteers, were spiked with EBOV Makona strain to achieve a final titer of 10^5^ fluorescent focus unit (FFU)/mL; then, the samples were stored at 37, 23 and 4 °C and at −80 °C as a control. Infective virus titer was determined at indicated time points (0, 12, 24, 48, 72, 96, and 120 h) in all specimens using a fluorescent focus-forming assay. Virus viability was most stable in EDTA-whole blood (Fig. 1a) and plasma (Fig. 1b) with titer reductions of 1.56 and 1.42 log10, respectively, at 37 °C after 120 h. The virus also retained infectivity at 23 °C in both of these sample specimen types. In contrast, virus titers in simulated capillary blood showed a substantial reduction when stored at 37 °C (Fig. 1c) and lost all infectivity by 96 h. This sample type also showed an extensive (1.98 log10) reduction in infectivity at 23 °C. EBOV was found to be the most unstable in urine at 37 °C, in which the virus lost infectivity completely by 72–96 h (Fig. 1d). Infectivity in urine decreased by 1.27 log10 at 23 °C during the course of the 5-day storage period. The results from semen highlighted a critical time window within the first 24 h at 37 °C, during which the virus lost most of its infectivity. Thereafter, a slower decay in infectivity was observed with complete loss of viability by 120 h (Fig. 1e). The titer in RPMI-1640 cell culture media showed a notable reduction of 3.95 log10 at 37 °C and 1.25 log10 at 23 °C after 120 h (Fig. 1f). The virus remained stable at 4 °C for all specimens.

The influence of repeated freeze–thaw cycles on virus infectivity was also tested. While one freeze–thaw cycle had no significant effect on virus infectivity, three freeze–thaw cycles exhibited moderate effects on plasma and urine samples with 0.29 and 0.62 log10 reductions, respectively, compared to the control (incubation in RPMI medium; 0.45 log10 reduction). In the case of EDTA-whole blood, an overall 1.11 log10 decrease in virus infectivity was observed after 3 cycles of freezing and thawing (data not shown).

Comparison of the initial and post-storage RNA quantity by real-time reverse transcription-polymerase chain reaction (RT-PCR) did not show significant virus RNA reduction under any of the tested conditions (temperature, clinical specimen type or different donors) during the 5-day storage period (*P* = 0.71).

Statistical analysis of the obtained data showed that temperature had a significant effect on virus viability (*P* < 0.001) with accelerated decay at 37 °C. According to our results, virus infectivity also decays with different kinetics at different temperatures in different specimens. Different types of the specimens showed a significant influence on virus viability at 37 °C (*P* < 0.001) and 23 °C (*P* = 0.005) but no significant effect at 4 °C (*P* = 0.269), confirming that EBOV is relatively stable and that this stability was independent of the matrix at low temperature. Samples from different donors showed no significant effect (*P* = 0.968) during the 5-day storage period. The results of semen samples were not included in the statistical analysis because of the low number of replicates due to cytotoxic effects on cell culture.

**Fig. 2:**
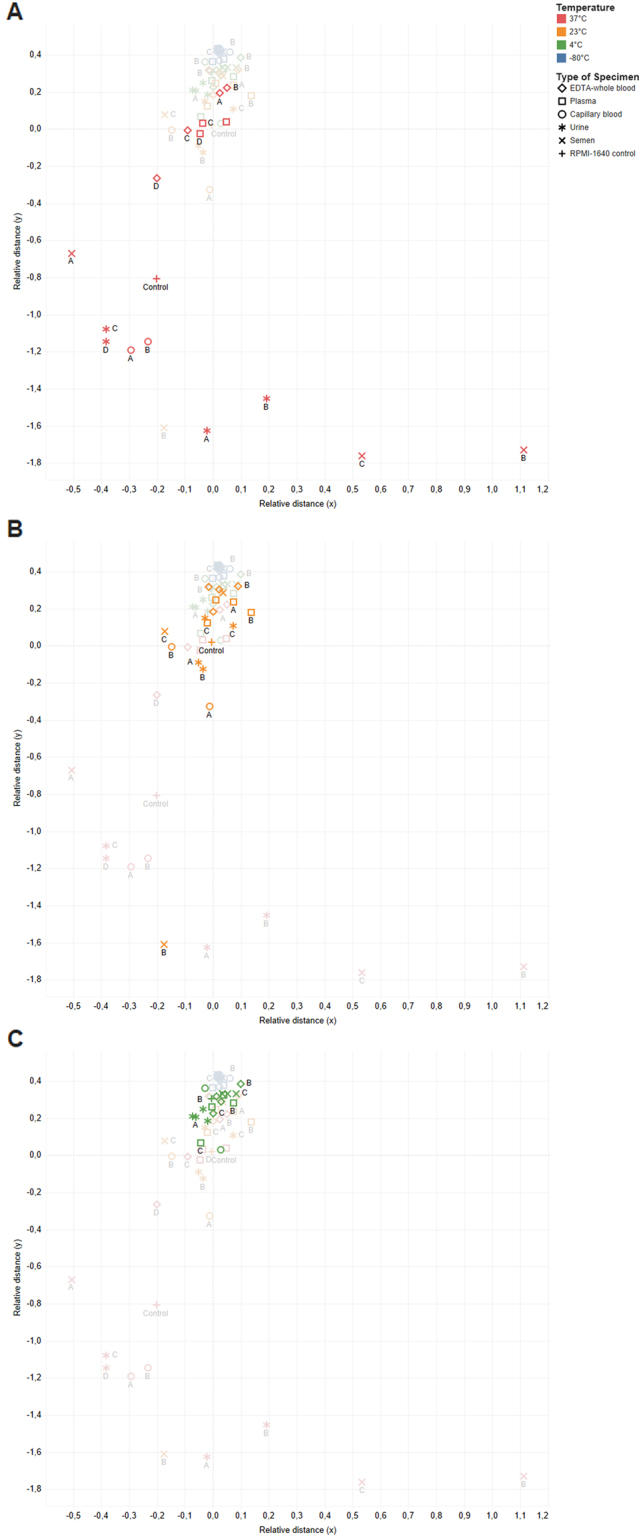
Results of the clustering algorithm for visual comparison of Ebola virus infectivity at different storage temperatures. **a** 37 °C, **b** 23 °C and **c** 4 °C. Data points are classified by color in the figures for different storage temperatures (as indicated). Different types of specimens are represented by different marker shapes (as indicated), and the four donors are indicated by letters

Clustering was further used to visualize the effects of temperature on virus titer (Fig. 2). The results of cluster analysis confirmed that the primary factor affecting virus infectivity was storage temperature with the 37 °C data points clustering separately from the other data points (Fig. 2a). Small sub-clusters by specimen type were also observed at 37 °C and 23 °C (Fig. 2b), but scattering was more prominent at 37 °C. Data points for samples at 4 °C formed a cluster without additional sub-cluster formation based on the specimen type (Fig. 2c). Additionally, no cluster formation based on different individual donors (indicated by letters) was observed at any temperature, leading to the conclusion that individual donor differences had no effect on virus infectivity decay.

## Discussion

The unprecedented scale of the 2013–2016 Ebola epidemic in West Africa highlighted several difficulties that were encountered during the outbreak response; long transport times and inappropriate transport and storage conditions for clinical specimens were identified among them^1^. Studies determining the stability of the virus RNA are important for diagnostic aspects, but the lack of viral isolation and cultivation as a confirmation of virus viability in EBOV RNA-positive samples is a limitation^3,11^. Given the observed persistence and shedding of EBOV long after recovery from disease, such knowledge is of critical importance.

The aim of our study was to assess factors that may affect virus isolation efficiency due to inappropriate shipping or storage conditions, which may impact longitudinal studies examining the infectiousness of different body fluids and clinical samples from EVD survivors. Moreover, virus isolation is important to study virus persistence and intra-host phenotypic changes and for various immunological studies. After sampling, samples in the field are often transported and/or stored at approximately 4 °C until further processing or short term at room temperature in Africa; room temperatures can vary between 20 and 40 °C depending on the time of day and season^16^. In addition, samples often undergo several freeze–thaw cycles during storage and transportation. To examine the variation in virus infectivity in five clinically relevant specimens, storage temperatures of 37, 23, 4 and −80 °C (control) were assessed.

Whole blood and plasma are recommended for the detection of EBOV; hence, most diagnostic tests are validated for these two types of specimens^17,18^. Our results showed that the virus maintains long-term infectivity in whole blood and plasma; infectivity decay in these samples was less than 1.5 log10 after 120 h, even at higher temperatures. Similar results have been described by Fischer et al.^11^. This prolonged infectivity could be due to the protective role of proteins in whole blood or plasma samples^19^. In one long-term stability study, EBOV suspended in guinea pig sera could be isolated for up to 46 days^20^. However, Prescott et al.^21^ investigated the postmortem stability of EBOV and found that the virus can be isolated from blood obtained from the body cavity of killed cynomolgus macaques for up to only 9 days. Venous blood sampling requires trained and qualified medical personnel and is difficult to perform on newborns and infants or people with religious convictions prohibiting such medical interventions. Capillary blood sampling is less invasive, has only minimal risk of needle stick injuries and can be useful for the diagnosis of patients with suspected EVD^7^. Therefore, as an alternative to whole blood, we aimed to determine the suitability of simulated capillary blood samples absorbed onto swab devices for virus isolation. The virus completely lost infectivity at 37 °C by 96–120 h and showed a 2.84 log10 reduction of infectivity at 23 °C by 120 h. This rapid loss of infectivity may be due to the rapid drying process, which is enhanced by the material of the swab applicator device. According to different environmental studies, studies on the viability of EBOV after drying blood on different surfaces led to disparate results. The average loss of infectivity ranged from 1 to 7 log10 reduction within 24–96 h at 21 °C depending on the surface and on the relative humidity, which appears to have an impact on virus viability, especially at higher temperatures^4,9,20,22^.

Among clinical specimens, urine samples can be useful for monitoring disease progress and are easy to collect^8,23^. However, urine samples are one of the body fluids that is most affected by storage, particularly when they are stored at 37 °C. Our study is the first to demonstrate the critical time window of 48–96 h for virus isolation from urine samples at higher temperatures. Notably, according to our data, infective virus titer can also decrease substantially within the first 12–24 h after sampling, depending on storage temperature. The low amount of protein and the presence of bacteria, proteases and RNases may contribute to the increased decay of virus infectivity^3^. Moreover, the presence of chemical components, such as ammonia, urea and a low pH, may have an additional impact, resulting in rapid virus degradation^24,25^.

According to Sissoko et al.,^14^ infectious EBOV can be detected in the seminal fluids of male survivors for up to 233 days, emphasizing the potential for sexual transmission. However, the effect of short-term storage at different temperatures on EBOV viability in semen has not been previously evaluated. Similar to urine and capillary blood, complete infectivity loss was observed in semen at 120 h when stored at 37 °C. Again, this result may be due to the presence of bacteria and proteolytic enzymes, which could have an effect on the decay rate. A slower reduction in infectivity was found at 23 °C with 2 log10 reduction, strengthening the findings published by Fischer et al^26^.

Our results showed that virus recovery from cell culture media was also substantially higher when the samples were stored at 4 °C than at higher temperatures, which is in agreement with the previous work of Piercy et al.^20^. Because the virus remains infective at room temperature or lower temperatures for longer than 120 h in some clinical specimen types, additional experiments are needed to determine the prolonged time period until the complete loss of virus infectivity. Moreover, EBOV strains/isolates could differ in their decay rates under different environmental conditions; therefore, more studies are needed to reveal these variations and determine the underlying biological mechanisms. Additionally, dried blood spot analysis could be a useful alternative for safely collecting, shipping and storing EVD blood samples, especially during outbreak emergencies during which sample logistics can be a major challenge. Finally, the fluorescent focus assay has not been validated for EBOV in clinical specimens; thus far, this method has been used in only simulated clinical specimens to determine virus infectivity. To implement this method for clinical diagnostic purposes, further validation of the assay is necessary. Statistical analysis of our data clearly demonstrated that temperature significantly affected virus infectivity, and thus inappropriate storage conditions, such as storage at 37 °C, reduced the efficacy of virus isolation. Further, specimen type had a significant effect on virus viability at 37 and 23 °C, which is important to consider when transporting clinical samples. The clustering method used for visual analysis confirmed that degradation of the Ebola virus was accelerated at 37 °C and that virus infectivity decay followed substantially different kinetics in distinct specimen types. Fischer et al.^11^ demonstrated that EBOV strain Makona viability decay in blood was independent of the environment, which is in agreement with our findings. However, we observed significant infectivity loss in capillary blood samples. Only minimal virus degradation was observed at 4 °C, confirming that EBOV is relatively stable and independent of the matrix at low temperatures. According to analysis of variance (ANOVA) and cluster analysis, individual donor differences showed no significant effect on virus viability during short-term storage.

Previously, no information was available concerning the effects of repeated freeze–thaw cycles on the degradation of EBOV in different clinically relevant sample specimens. Our results show that the most affected body fluid was EDTA-whole blood with a 1.11 log10 reduction in virus infectivity. Repeated freezing and thawing may have caused structural damage to the virion, thereby reducing virus viability^27^.

During the 2013–2016 West African Ebola outbreak, the criterion to discharge convalescent patients from Ebola treatment centers was two consecutive EBOV PCR negative blood samples^28^. Nonetheless, some EVD patients remained real-time RT-PCR positive in different body fluids, such as urine, sweat, feces and semen, with high Ct values that indicated potentially low infective viral loads^29^. Although these sample types are critical to estimating the risk of infectivity and virus transmission by providing valuable information about the shedding of contagious EBOV into body fluids during the acute and convalescent phase of EVD patients, they are also subjected to the greatest degradation of EBOV during inappropriate storage conditions. In conclusion, our study highlights the need for proper sample storage (i.e., at 4 °C) during transportation and to avoid repeated thawing and refreezing during short- and long-term storage of samples in which virus isolation will be performed.

## Materials and methods

### Virus stock propagation

Vero E6 cells were used for in vitro propagation and titration of the Ebola virus/*Homo sapiens*-tc/GIN/2014/Makona-WPGC05 strain (kindly provided by Stephan Günther, Bernhard Nocht Institute Hamburg, Germany). Cells were grown in RPMI-1640 (Sigma-Aldrich, St. Louis, MO, USA) cell culture media supplemented with 5% fetal bovine serum (BioSera, Nuaillé, France), penicillin and streptomycin (Sigma-Aldrich) and were maintained as adherent cell lines at 37 °C and 5% CO_2_. The infected cell culture supernatant was collected 5 days post infection, centrifuged at 3500 rpm for 20 min at 4 °C and aliquoted for storage at −80 °C until further use. All work with infectious Ebola virus was performed at the National Public Health Institute, National Biosafety Laboratory in Hungary under biosafety level 4 (BSL-4) conditions.

### Sample preparation

Ebola virus viability was determined in five different types of diagnostically relevant specimens, including EDTA-whole blood, plasma, simulated capillary blood, urine and semen, obtained from healthy volunteers. RPMI-1640 cell culture media supplemented with penicillin and streptomycin were also tested. EDTA-whole blood was collected by venipuncture from four volunteers using BD Vacutainer K2E (Becton-Dickinson, Franklin Lakes, NJ, USA), and the plasma was obtained by centrifugation of EDTA-whole blood at 3500 rpm for 10 min. To simulate capillary blood samples, 50 µL of EDTA-whole blood from two individuals was transferred to and absorbed on swab collection applicators (Biolab, Budapest, Hungary). The applicators were placed back into their cases for storage and swirled into 500 µL phosphate-buffered saline prior to testing. Urine samples obtained from four volunteers and semen samples from three male donors were centrifuged at 3500 rpm for 10 min before use in the experiment. All samples were tested for cytotoxicity, bacterial and fungal contamination and diluted 1:10 in RPMI-1640 cell culture media.

### Storage conditions

Two replicate samples of each type were spiked with EBOV to achieve a final titer of 10^5^ FFU/mL after accounting for inhibitory effect of the sample on the FFU assay. The samples were then aliquoted and stored at different temperatures (37, 23, 4 and −80 °C) for 0, 12, 24, 48, 72, 96 or 120 h. At the indicated time points, aliquots of each sample were collected and stored at −80 °C until titration at the end of the experiment. The influence of repeated freeze–thaw cycles on virus infectivity was also tested. Aliquots of EDTA-whole blood, plasma, urine and RPMI-1640 samples were frozen to −80 °C for 1 h, then thawed and stored at 23 °C for another hour. The freeze–thaw cycle was then repeated two additional times. The titer of the virus was determined after each freeze–thaw cycle.

### Molecular methods

Viral RNA was extracted using the Roche High Pure Viral RNA Kit (Roche, Basel, Switzerland) following the manufacturer’s instructions but with an additional wash step (with Wash Buffer) to reduce potential PCR inhibitors. Real-time RT-PCR was conducted using Roche LightCycler 480 RNA Master Hydrolysis Probes (Roche) reagents with primers and probes targeting the GP gene of the Ebola virus on the LightCycler 2.0 (Roche) platform^30^.

### Determination of infective virus titer by fluorescent focus-forming assay

After the indicated storage period, each sample was titrated in triplicate in 96-well plates according to the protocol described by Canakoglu et al.^31^ with minor modifications. Quantification of the virus titer was performed using a fluorescence microscope and is expressed in FFU/mL. In the case of end-point samples, the absolute infectivity loss was also confirmed by virus isolation using three blind passages on cell culture.

### Compliance with ethical standards

Informed consent to publish was obtained from all volunteers included in this study.

### Statistical analysis

Statistical analysis was performed with the Statistica Software package (Statsoft, Budapest, Hungary) using ANOVA to identify factors (i.e., temperature, type of specimen and different donors) that may impact virus viability during shipping or storage. The results were deemed to be significant with *P* < 0.05. Decay rates derived from the slopes of the fitted linear curves were estimated using the ordinary least squares method.

A clustering algorithm was applied for visual comparison of the observed factors of temperature, type of specimen and donor using Tableau Software (Tableau, Seattle, WA, USA) with relative distances calculated using the Euclidean distance method.
